# Anti-Melanogenic Activity of *Calocedrus formosana* Wood Essential Oil and Its Chemical Composition Analysis

**DOI:** 10.3390/plants11010062

**Published:** 2021-12-25

**Authors:** Wen-Wei Hsiao, K. J. Senthil Kumar, Hui-Ju Lee, Nai-Wen Tsao, Sheng-Yang Wang

**Affiliations:** 1 Experimental Forest, College of Bio-Resources and Agriculture, National Taiwan University, Taipei 10617, Taiwan; hsiaoww@gmail.com; 2 Bachelor Program of Biotechnology, National Chung Hsing University, Taichung 40227, Taiwan; zenkumar@dragon.nchu.edu.tw; 3 Department of Forestry, National Chung Hsing University, Taichung 40227, Taiwan; ab840909@gmail.com (H.-J.L.); selina761108@hotmail.com (N.-W.T.); 4 Agricultural Biotechnology Research Center, Academia Sinica, Taipei 11574, Taiwan

**Keywords:** *Calocedrus formosana*, Cupressaceae, essential oil, anti-melanogenesis, tyrosinase

## Abstract

*Calocedrus formosana* (Cupressaceae) is one of the five precious woods of Taiwan. In this study, we investigated the anti-melanogenic activity of *C. formosana* wood essential oil (CFEO) and its bioactive components in vitro. Initially, CFEO exhibited strong mushroom tyrosinase activity in the cell-free mushroom tyrosinase assay system with an IC_50_ value of 2.72 µg/mL. Next, treatment with CFEO significantly as well as dose-dependently reduced a combination of α-melanocyte-stimulating hormone and forskolin (α-MSH-FSK)-induced melanin synthesis in B16-F10 cells. Indeed, 80 μg/mL CFEO completely inhibited melanin production, which is similar to that of control cells. Further studies revealed that treatment with CFEO significantly inhibited melanogenesis regulatory proteins, including TRP-1, TRP-2, and MITF, whereas tyrosinase was unaffected by either α-MSH-FSK or CFEO. In addition, the composition of the CFEO was characterized. The major components of CFEO were α-terpineol (23.47%), shonanic acid (10.45%), terpinen-4-ol (12.23%), thymol (5.3%), piperitone (3.44%), berbenone (2.81%), thujic acid (1.65%), and chaminic acid (0.13%). Among them, shonanic acid (**1**), thujic acid (**2**), and chaminic acid (**3**) were uncommon constitutes in essential oils, which could be the index compounds of CFEO, and the structure of these compounds were confirmed by spectral analysis. Furthermore, we found that thymol is an active ingredient responsible for CFEO’s anti-melanogenic activity. Based on these results, we suggest that CFEO or thymol could be a potential candidate for the development of skin whitening products for cosmetic purposes.

## 1. Introduction

Melanin is a group of natural pigments found in most living organisms. Cellular melanin is biosynthesized through a complicated process, called melanogenesis, which takes place in the melanosome, an intracellular organelle found within the melanocytes. The melanin-containing melanosomes are transported to the neighboring keratinocytes in the epidermis. Based on melanin concentration in the epidermis, skin color and shade were determined [1]. Cutaneous melanin plays a functional role in the epidermal homeostasis, is important for responding to and protecting the skin from the environmental stress, including ultraviolet radiation from the sunlight. However, abnormal melanin production leads to various dermatological disorders, such as freckles, solar lentigo, melasma, vitiligo, melanoma, and other hyper pigmentary skin diseases [2]. Therefore, regulating melanogenesis is one of the desirable strategies to control hyperpigmentary skin diseases and beauty-enhancing cosmetic purposes. Indeed, the development of anti-melanogenic agents are not only important for curing hyperpigmentation for cosmetic, pharamaceutical, and medicinal purposes; this is also effective in melanoma therapy, in which melanogenesis and the concentration of melanin level can affect chemo-/radiotherapies and the survival period of patients with melanoma [3].

The types and amounts of melanin production in melanocytes were determined by various endogenous factors (hormonal changes, stem cell factors, inflammation, and aging) and exogenous factors (repetitive UV exposure, pathogenic infection, environmental pollution, chemical stimuli, and physical damage) [4]. Upon stimulation by either extrinsic or intrinsic factors, melanocytes produce melanin. There are three key enzymatic components playing a central role in melanin biosynthesis: tyrosinase (TYR), tyrosinase-related protein-1 (TRP-1), and dopachrome tautomerase/tyrosinase-related protein-2 (DCT/TRP-2). Microphthalmia-associated transcription factor (MITF) is a basic helix leucine zipper transcription factor regulating tyrosinase and tyrosinase-related family genes in melanin producing cells. Tyrosinase, a copper-containing enzyme, catalyzes two rate-limiting reactions during melanin biosynthesis, including hydroxylation of _L_-tyrosinse into _L_-3,4-dihydroxyphenylalanine (_L_-DOPA) and further oxidation of _L_-DOPA into DOPA quinone, whereas TRP-1 and TRP-2 play a crucial role in catalyzing eumelanin-producing reactions. TRP-2 catalyze DOPA chrome into 5,6-dihydroxyindole (DHI) or 5,6-dihydroxyindole-2-carboxylic acid (DHICA). In the final step, DHI and DHICA are oxidized by TRP-1 and leading to melanin production [5].

Since tyrosinase is a key player in melanogenesis, tyrosinase inhibition is a common strategy for the development of anti-melanogenic agents. Now, there are two major strategies are following to identify potential anti-melanogenic agents: They are the therapeutic agent directly inhibits tyrosinase enzyme activity or modulates cellular signaling cascades involved in melanin biosynthesis [6]. Numerous tyrosinase inhibitors from synthetic or natural sources, including polyphenols, terpinoids, benzoate derivatives, long-chain fatty acids, and sterols have been identified [7]. However, few tyrosinase inhibitors have been withdrawn from medical and cosmetic usage due to their toxicity or severe side-effects [7]. Owing to safety concerns, it is necessary to identify an alternative and natural tyrosinase inhibitor or melanin biosynthesis pathway modulator without producing adverse side-effects.

Essential oils, also known as plant volatiles, are widely incorporated into modern cosmetic products due to their complexity of active ingredients, strong fragrance, and attractive marketing image [8]. In recent years, essential oils and their components are gaining public interest because of wide-spread consumer acceptance and broad-spectrum of functional use. Accumulating scientific evidence proved that essential oils can be used to treat various skin disorders, including acne, pre-mature aging, hyperpigmentation, as well as protecting skin from UV radiation. It has been reported that Origanum (*O. syriacum* and *O. ehrenbergii*) essential oils [9], leaf essential oils of *Alpinia zerumbet* [10], *Melaleuca quinquenervia* [11], leaf and rhizome essential oils of *Alpinia nantoensis* [12], flower essential oils of *Eucalyptus camaldulensis* [13], stem bark essential oils of *Cinnamomum cassia* [14] exhibited strong anti-melanogenic activity in vitro.

Taiwan incense cedar, *Calocedrus formosana* (syn. *C. macrolepis* var. *formosana*) belonging to the Cupressaceae family, has been used to make incense since ancient times in Taiwan. More than 100 compounds have been isolated from the plant, including monoterpenoids, diterpenoids, lignans, and steroids [15,16]. The compounds of *C. formosana* possessed the diverse activities, such as anti-oxidative [17,18], anti-termitic and anti-fungal [19], anti-inflammatory [20], and anti-cancer [21] properties. However, so far, there have been no reports regarding the potential dermatological application of wood essential oils of *C. formosana*. The aim of this study is to determine the chemical compositions of the wood essential oils of *C. formosana* (CFEO) and to determine its anti-melanogenic activity in vitro.

## 2. Results

### 2.1. Wood Essential Oil of Calocedrus formosana (CFEO) Inhibits Mushroom Tyrosinase Actvity

**Figure 1:**
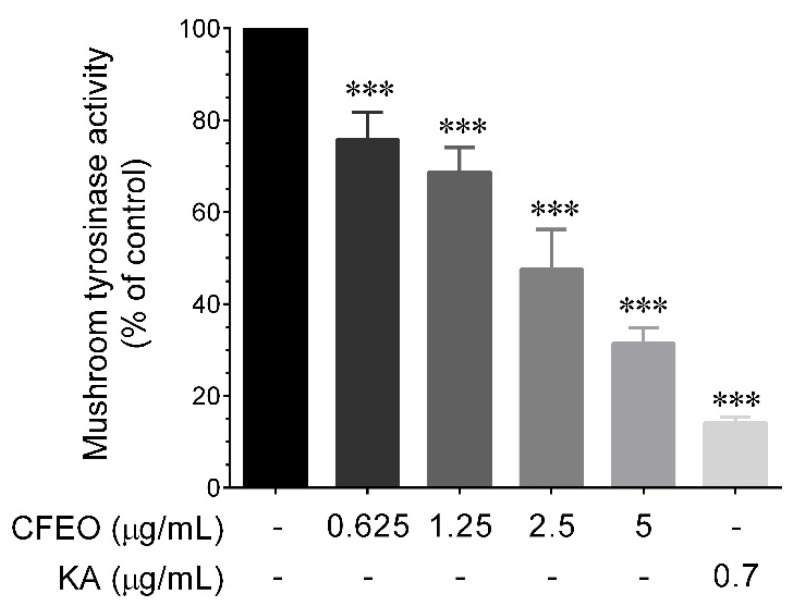
Effect of CFEO on mushroom tyrosinase activity. The data are represented as mean ± SD of three indipendent experiments. Statistical significance was set as *** *p* < 0.001 compared with sample treatment groups vs. control group.

Tyrosinase is a rate-limiting enzyme involved in melanin production. The reaction rate of tyrosinase that converts _L_-DOPA into DOPA quinone can be used to evaluate the activity of tyrosinase, which in turn can be used as a screening platform for anti-melanogenic agents. The anti-tyrosinase activity of CFEO was examined based on previously described cell-free mushroom tyrosinase inhibitory assay along with _L_-DOPA as a substrate [12]. As shown in Figure 1, incubation of mushroom tyrosinase with increasing concentrations of CFEO (0.625–5 μg/mL) significantly as well as dose-dependently reduced mushroom tyrosinase activity with an IC_50_ value of 2.72 μg/mL. Indeed, compared with control group (without test samples; 100%), incubation with 0.625, 1.25, 2.5, and 5 μg/mL CFEO reduced mushroom tyrosinase activity to 75.8%, 68.7%, 47.6%, and 31.4%, respectively. In addition, the well-known natural tyrosinase inhibitor, kojic acid (KA) reduced mushroom tyrosinase activity to 14.2%. This data gave positive feedback that CFEO could have the anti-melanogenic property in vitro.

### 2.2. CFEO Inhibits α-MSH-FSK-Induced Melanin Biosynthesis in B16-F10 Cells

**Figure 2:**
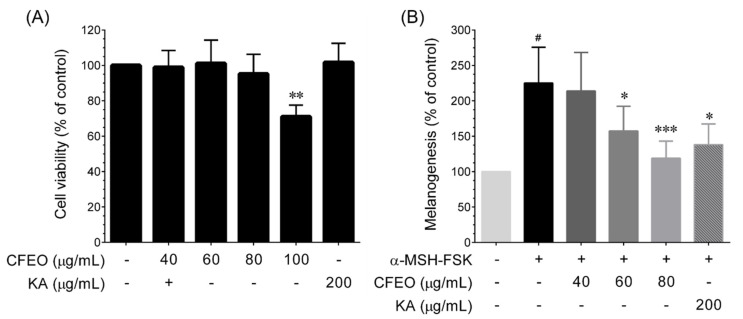
Effect of CFEO on α-MSH-FSK-induced melanin in B16-F10 cells. (**A**) Cells were incubated with increasing concentrations of CFEO (40–100 μg/mL) or KA (200 μg/mL) for 72 h. Cell viability was determined by MTT colorimetric assay. Data represent the mean ± SD of three independent experiments. Statistical significance was set as ** *p* < 0.01 compared with sample treatment groups vs. control group. (**B**) Cells were treated with increasing concentrations of CFEO or KA and stimulated with α-MSH-FSK for 72 h. The cellular melanin content was determined by colorimetric assay. Data represent the mean ± SD of three independent experiments. Statistical significance was set as ^#^ *p* < 0.01 compared with control vs. α-MSH-FSK and * *p* < 0.05, and *** *p* < 0.001 were significant between sample treatment groups vs. α-MSH-FSK treatment group.

Prior to examining the anti-melanogenic activity of CFEO, the cytotoxic effect of CFEO was examined by MTT colorimetric assay. B16-F10 cells were treated with various concentrations of CFEO for 72 h and then the cytotoxicity was determined. As shown in Figure 2A, treatment with CFEO does not show significant cytotoxicity towards B16-F10 cells up to a concentration of 80 μg/mL, whereas a significant reduction in cell viability was observed over a dose of 80 μg/mL. In addition, treatment with 200 μg/mL KA does not produced cytotoxicity against B16-F10 cells, indicating that CFEO (up to 80 μg/mL) and KA (200 μg/mL) were not cytotoxic to the B16-F10 cells. Forskolin (FSK) and α-melanocyte stimulating hormone (α-MSH) were characterized as potent melanin inducers in vitro [20]. In this study, a combination of α-MSH (100 μM) and FSK (20 µM) was used to stimulate melanin biosynthesis in B16-F10 cells and the inhibitory effect of CFEO was examined. As shown in Figure 2B, exposure of B16-F10 cells to α-MSH-FSK for 72 h remarkably induced melanin production, which was nearly 2-fold higher than that of the control group. Interestingly, treatment with CFEO significantly as well as dose-dependently inhibited α-MSH-FSK -induced melanin production with an IC_50_ value of 75.23 μg/mL. The inhibition ratio of CFEO of 40 and 60 µg/mL were 31.3% and 54.5%, respectively, whereas treatment with 80 μg/mL CFEO almost completely inhibited melanin production. In addition, the melanin inhibitory effect of 80 μg/mL CFEO is highly comparable with 200 μg/mL KA, a known melanin inhibitor. The result indicated that CFEO has high potential to inhibit melanin production and can be considered as a suitable source for the commercial production of skin whitening agent.

### 2.3. CFEO Inhibits Melanogeneis Regulatory Proteins in B16-F10 Cells

**Figure 3:**
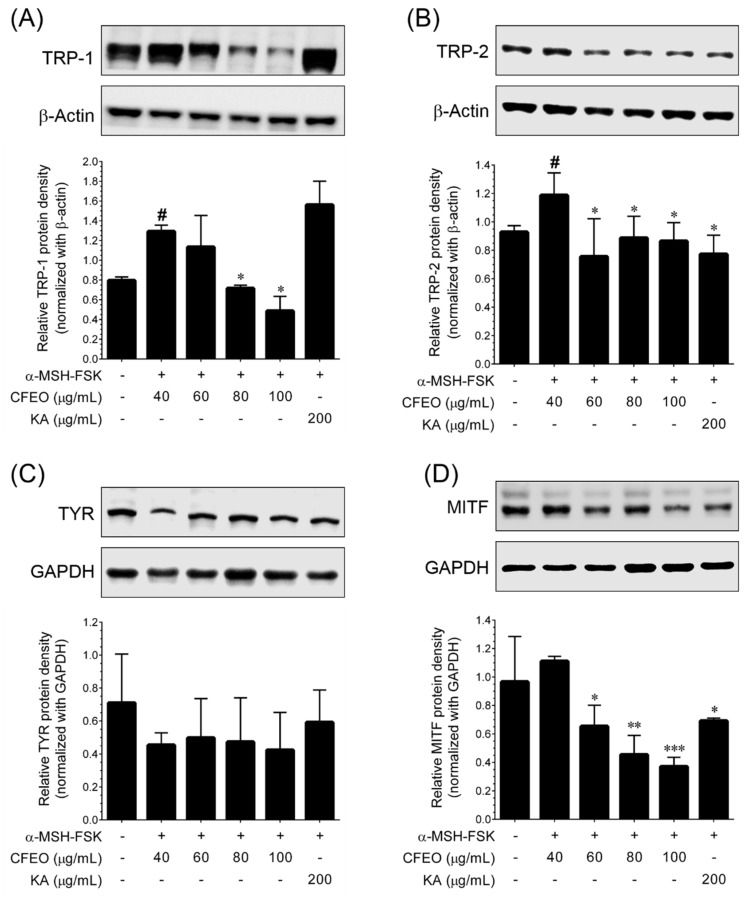
Effect of CFEO on α-MSH-FSK -induced melanogenesis regulatory proteins in B16-F10 cells. Cells were treated with increasing concentrations of CFEO or KA and stimulated with α-MSH-FSK for 72 h and (**A**), TRP-1 (**B**), TRP-2 (**C**), and MITF (**D**) protein expression levels were determined by Western blotting. Target protein bands were normalized with the internal controls either β-actin or GAPDH. Data represent the mean ± SD of three independent experiments. Statistical significance between control vs. α-MSH-FSK group was set as ^#^ *p* < 0.01. Significance between sample treatment groups vs. α-MSH-FSK treatment group were set as * *p* < 0.05, ** *p* < 0.01, and *** *p* < 0.001.

Melanin production is critically regulated by tyrosinase (TYR), TYR-related protein-1 (TRP-1), and TRP-2. Upon stimulation, the increased expression levels of these proteins were directly proportional to the melanin production. Therefore, we sought to examine whether CFEO-mediated reduction in melanin production could possibly be associated with downregulation of melanogenic regulatory proteins, including TYR, TRP-1, and TRP-2. We found that α-MSH-FSK treatment remarkably elevated TRP-1 expression, whereas CFEO treatment significantly as well as dose-dependently inhibited TRP-1 expression. Interestingly, when compared with control group, treatment with 80 μg/mL CFEO decreased TRP-1 below to the basal. Notably, treatment with KA failed to inhibit TRP-1 under similar stimulated condition (Figure 3A). A similar pattern of inhibition of TRP-2 was found in CFEO treated cells (Figure 3B). Interestingly, treatment with KA also significantly inhibited TRP-2 expression. In contrast, α-MSH-FSK exposure failed to upregulate TYR expression in B16-F10 cells, in contrast, treatment with CFEO significantly and dose-dependently increased TYR expression (Figure 3C). These results suggest that CFEO-mediated inhibition of melanin production was caused by downregulation of TRP-1.

MITF regulates TRP-1, TRP-2, and TYR expression in melanin producing cells. Therefore, next we examined whether CFEO-mediated inhibition of TRP-1 expression was associated with transcriptional inhibition of MITF. The MITF protein expression was measured after exposed to α-MSH-FSK in the presence or absence of CFEO. According to the result shown in Figure 3D, a significant increase in MITF protein levels was observed after exposed to α-MSH-FSK, whereas CFEO treatment significantly as well as dose-dependently reduced the MITF expression. Indeed, all the concentrations of CFEO reduced MITF expression below to the basal level. In addition, the MITF inhibitory effect of CFEO was much stronger than that of KA. Taken together, these data provided a strong basis that CFEO could inhibit melanin production possibly downregulating TRP-1 via MITF.

### 2.4. Chemical Composition of CFEO

**Figure 4:**
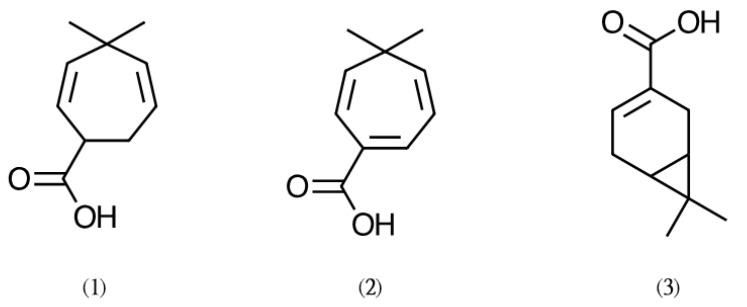
Chemical structures of three index compounds (**1**) Shonanic acid, (**2**) Thujic acid, and (**3**) Chaminic acid.

The yields of CFEO by hydro-distillation was 2.01% (*w/w*). The major chemical constituents of CFEO were determined by GC–MS analysis. The relative contents (%) of major compounds in CFEO is shown in Table 1. Regard to the three absorption peaks in GC analysis, which are located at the retention time of 29.29 (**1**), 31.31 (**2**), and 32.00 min (**3**), which are not identified by standard databases, including Wiley/NBS, NIST, and Kovats indices. These tree compounds were further isolated and purified by column chromatographic and high-performance liquid chromatographic (HPLC); and analyzed NMR and mass spectrometry (MS). They were identified as shonanic acid (**1**), thujic acid (**2**), and chaminic acid (**3**) (Figure 4). A total of 24 volatile compounds were identified in CFEO, comprising 78.86%. Among them, α-terpineol (23.47%), terpinen-4-ol (12.23%), γ-terpineol (6.6%), thymol (5.3%), piperitone (3.44%), and berbenone (2.81%) are the major components, which made up around 53.85% of the content of the CFEO.

### 2.5. Effects of CFEO Major Constituents on Melanin Production

**Figure 5:**
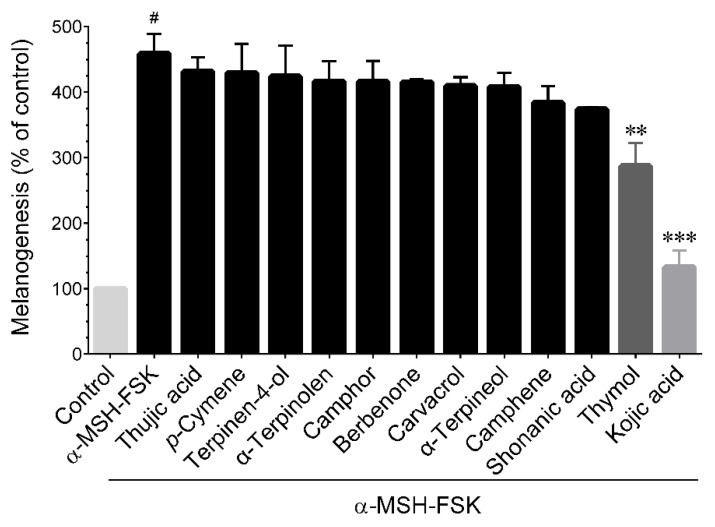
Effect of major compounds from CFEO on α-MSH-FSK-induced melanin production in B16-F10 cells. Cells were incubated with 200 μM of indicated compounds or 1400 μM KA and induced with α-MSH-FSK for 72 h. The cellular melanin content was determined by colorimetric assay. Data represent the mean ± SD of three independent experiments. Statistical significance between control vs. α-MSH-FSK group was set as ^#^ *p* < 0.01. Significance between sample treatment groups vs. α-MSH-FSK treatment group were set as ** *p* < 0.01 and *** *p* < 0.001.

**Figure 6:**
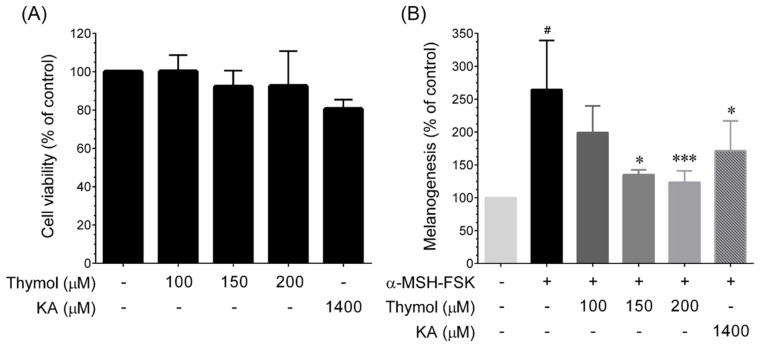
Effect of thymol on melanin inhibition. (**A**) Cells were incubated with increasing doses of thymol (100, 150, and 200 μM) or KA (1400 μM) for 72 h. Cell viability was determined by MTT colorimetric assay. Data represent the mean ± SD of three independent experiments. (**B**) Cells were treated with increasing concentrations of thymol or KA and stimulated with α-MSH-FSK for 72 h. The cellular melanin content was determined by colorimetric assay. Data represent the mean ± SD of three independent experiments. Statistical significance was set as ^#^ *p* < 0.01 compared with control vs. α-MSH-FSK and * *p* < 0.05, and *** *p* < 0.001 compared with sample treatment groups vs. α-MSH-FSK treatment group.

In order to identify the active ingredients in CFEO, the main compounds of essential oils including camphene, α-terpineol, carvacrol, camphor, terpinen-4-ol, α-terpinolen, *p*-cymene, berbenone, thymol, thujic acid, and shonanic acid were evaluate the bioactivity on the alteration of melanogenesis. Prior to examine the anti-melanogenic activity of CFEO’s constituents, the MTT assay was conducted to evaluate the cytotoxicity of CFEO compounds. The result revealed that no cytotoxicity effect on B16-F10 cells below the dosage at 200 μM of CFEO’s major compounds and 1400 μM of kojic acid (data not shown). Figure 5 shows the melanin inhibitory activity of major components of CFEO. It indicated that only 200 μM thymol could inhibit melanin production, and its activity was equivalent to 1400 μM KA did. As shown in Figure 6A, thymol does not show any cytotoxicity against B16-F10 cells up to a dose of 200 μM. To determine the IC_50_ of thymol on the inhibition of melanin production, different concentrations of thymol were used to examine the anti-melanin production in α-MSH-FSK-stimulated B16-F10 cells (Figure 6B). The result indicated that the amount of IC_50_ thymol needed to inhibit melanin production in B16F10 cells is approximately 160 μM.

## 3. Discussion

To pursue beauty is human nature. However, people have a different opinion of beauty; many people from East Asian countries want white and flawless skin [22]. The World Health Organization (WHO) survey found that up to 40% of women in China, Malaysia, Philippines, and South Korea have experience in using whitening products [23]. According to a research by Global Industry Analyst, a US market research firm, the global skin whitening market sales in 2017 was USD 4.8 billion, and it is estimated to double in 2027 [24]. The whitening products in the market mostly contain acid ingredients, which have high cytotoxicity, insufficient penetrating power, and low activity and also often cause skin sensitivity and contact dermatitis for long-term application. Therefore, the development of safe, bio-combatable, and naturally derived skin whitening agents become the mainstream of whitening product development [25]. Essential oils provide the broad-spectrum of health benefits and preservatives, which is predominantly used in the preparation of pharmaceutical and cosmetic products since ancient time [26]. Moreover, the pleasant aroma of essential oils also permits to apply in various cosmetic product. Specifically, essential oils are well studied on their direct tyrosinase inhibitory activity, which is a key strategy for the development of skin-lightening agents [27]. *C. formosana* has been used to make the incense due to its pleasant aroma. Several studies have demonstrated that the extracts and derivate compounds of *C. formosana* have diverse activities. However, the bioactivity investment of *C. formosana* is rare. Therefore, we focused on the anti-melanogenic activity of CFEO in this study.

Inhibitory activity against mushroom tyrosinase is a primary method to determine the skin whitening efficacy of candidature agents, since tyrosinase enzyme plays a central role in melanin biosynthesis. Therefore, we utilized this assay to determine the mushroom tyrosinase inhibitory effect of CFEO. In comparable with a previous study by Aumeeruddy-Elalfi et al. [27] examined mushroom tyrosinase inhibitory efficacy of 19 essential oils. Among the studied essential oils, *Cinnamomum zeylanicum*, *Citrus grandis*, and *Citrus hystrix* exhibited strong tyrosinase inhibitory effects with an IC_50_ values of 2.05 μg/mL, 2.07 μg/mL, and 2.08 μg/mL, respectively, which were similar to that of kojic acid (IC_50_; 2.28 μg/mL). However, not all the essential oils possess direct tyrosinase inhibition. For example, our previous study has shown that leaf and rhizome essential oils of *Alpinia nantoensis* failed to inhibit mushroom tyrosinase activity [12]. In this study, we found that CFEO significantly as well as dose-dependently inhibited mushroom tyrosinase activity with an IC_50_ value of 2.72 μg/mL. According to the mushroom tyrosinase inhibitory assay, CFEO presented better inhibitory activity than several plant essential oils, indicating that CFEO has the potential to inhibit melanin production.

Melanin biosynthesis is orchestrated with several sequential steps, including receptor activation, production of intracellular cAMP, and transcriptional activation of MITF and transcription of tyrosinase family genes. Forskolin and α-MSH were identified as potent cAMP activators which trigger melanogenesis in vitro [28]; in this study, we induced melanin production through a combination of α-MSH and forskolin and determined the inhibitory effect of CFEO. We found that CFEO treatment significantly blocked melanin production in B16 cells, which is well correlated with other observations that essential oils from *Cinnamomum cassia, Melaleuca quinquenervia, Alpinia zerumbet*, *Alpinia nantoensis, Eucalyptus camaldulensis, Origanum syriacum,* and *Origanum ehrenbergii* showed strong melanin inhibition in a similar condition to in vitro experiments [9,10,11,12,13]. In addition, essential oils have the capability to inhibit melanogenesis through dual inhibitory mechanisms, including direct inhibition of tyrosinase enzyme activity and downregulating the melanin biosynthesis pathway through modulating cellular signaling cascades [14,29]. Tyrosinase family proteins, including TYR, TRP-1, and TRP-2 were the key players of melanin biosynthesis. In this study, we found that CFEO failed to modulate TYR expression, whereas TRP-1 and TRP-2 expression levels were significantly downregulated; the positive drug control KA does not affect the TRP-1 expression, which is well correlated with other observations [13]. Transcriptional and post-transciptional regulation of MITF is a hallmark event of melanogenesis. MITF can upregulate tyrosinase and its related proteins by binding to an M-box motif in their promoter site because MITF contains a basic helix-loop-helix-leucine zipper domain in its structure [30]. In this study, we found that CFEO treatment significantly reduced MITF expression and activity even better than kojic acid. That is, CFEO inhibited TRP-1 and TRP-2 expression then reduced the production of melanin through reduced MITF activity.

In this study, the chemical composition of CFEO was analyzed. GC-MS analysis resulted with 24 identified compounds in CFEO, most of these constituents are monoterpenoids. Three unique compounds in CFEO were isolated and identified from CFEO, namely shonanic acid, thujic acid, and chaminic acid. The main compounds of CFEO were evaluate the bioactivity on the alteration of melanogenesis. Among them, thymol exhibited the strongest the inhibitory melanin production activity, the IC_50_ of thymol on inhibiting melanin production in B16F10 cells is approximately 160 μM. A previous study further supports our finding that thymol strongly inhibits mushroom tyrosinase activity at both monophenalase (_L_-tyrosine oxidized and converts into _L_-DOPA) and diphenalase (_L_-DOPA converts into DOPA quinone/DOPA chrome) phase. The anti-oxidant property of tymol is characterized as a mechanism involved in the anti-melanogenic activity of thymol [31]. Escobar and his coworkers had pointed out thymol possessed a variety of biological activities [32]. Recently, it has been reported that the antioxidant activity of thymol could reduce the conversion of _L_-DOPA into dopaquinone by inhibiting the expression of TYR protein, thereby reducing the melanin induced by α-MSH [33]. Thus, the anti-melanogenesis principal of CFEO might be thymol.

## 4. Materials and Methods

### 4.1. Preparation of C. formosana Wood Essential Oil (CFEO)

Cut logs of a 35-year-old *C. formosana* were collected in June 2019 from Nantou County, Taiwan, and the species was identified by Prof. Sheng-Yang Wang, Department of Forestry, National Chung Hsing University, Taiwan. The voucher specimen was deposited in the herbarium of the same university. Air-dried wood chips were subjected to hydro-distillation in a Clevenger-type apparatus for 6 h, followed by determination of oil contents. The wood essential oils were stored in airtight sample vials prior to analysis of gas chromatography–mass spectrometry (GC–MS) and bioactivity evaluation.

### 4.2. Chemicals and Reagents

Roswell Park Memorial Institute 1640 (RPMI-1640) medium, fetal bovine serum (FBS), penicillin and streptomycin were obtained from Life Technologies, Grand Island, NY, USA. Mushroom tyrosinase (EC 1.14.18.1), α-MSH, _L_-DOPA, dimethyl sulfoxide (DMSO), and 3-(4,5-dimethyl-thiazol-2-yl)-2,5-diphenyl tetrazolium bromide (MTT) were purchased from Sigma-Aldrich (St Louis, CA, USA). Forskolin was obtained from Selleckchem (Houston, TX, USA). Antibodies against TYR, TRP-1, TRP-2, β-actin, and GAPDH were purchased from Santa-Cruz Biotechnology, Dallas, TX, USA. Antibody against MITF was obtained from Abcam, Cambridge, UK. Horseradish peroxidase (HRP)-linked anti-mouse IgG and anti-rabbit IgG anti-bodies were obtained from Cell Signaling Technology, Danvers, MA, USA. All other chemicals were reagent grade or HPLC grade and supplied by either Merck (Darmstadt, Germany) or Sigma-Aldrich.

### 4.3. Cell Culture and Cell Viability Assay

Murine melanoma (B16-F10) cell line was obtained from American Type Culture Collection (ATCC, Manassas, VA, USA). Cells were cultured in RPMI-1640 medium, supplemented with 10% FBS, glucose, penicillin and streptomycin. Cells were grown in 10 cm culture dish and incubated in a humidified atmosphere containing 5% CO_2_ at 37 °C. Cells were sub-cultured every three days. Cell viability was assessed by MTT colorimetric assay, as described previously [34]. In brief, 2 × 10^5^ cells were seeded onto a 24-well cell culture plate. After an overnight incubation, cells were treated with various doses of test samples for 72 h. All the test samples were dissolved in 0.1% DMSO and the control group has 0.1% DMSO. After the treatment, culture media was removed, and the cells were incubated with fresh media containing 0.5 mg/mL MTT for 2 h. The formazon crystals produced during the incubations was dissolved by 2 mL DMSO. The absorbance was measured at 570 nm using micro-plate reader (μQuant, Biotek Instruments, Winooski, VT, USA). The percentage of viable cells were calculated by the following formula: OD_570_ of sample treated cells/OD_570_ of control cells × 100.

### 4.4. Mushroom Tyrosinase Assay

Mushroom tyrosinase activity was determined by ELISA micro-plate reader method as described previously [35], with minor modifications. Briefly, a 300 μL reaction mixture containing 70 μL of 50 mM sodium phosphate buffer (pH 6.8), 10 μL of various concentrations of test samples, 20 μL of mushroom tyrosinase (100 IU), and 200 μL of substrate solution consisting of 4 mM _L_-DOPA in sodium phosphate buffer, was added into a 96-cell micro-plate and incubated in the dark for 10 min at room temperature. The absorbance of DOPA quinone formation was measured every 20 min for 3 min at 475 nm using a micro-plate reader (Biotek Instruments). Kojic acid was used as a positive drug control. To calculate the percentage of mushroom tyrosinase activity, the following formula was used: Sample treatment group/control group × 100.

### 4.5. Determination of Melanin Content

Melanin content was determined as described previously [12], with minor modifications. Briefly, 2 × 10^5^ cells were seeded onto a 6-well culture plate and incubated overnight to adhere the cells. The 50% confluent cells were treated with melanogenesis inducer (a mixture of 20 μM FSK and 100 nM α-MSH) or various doses of test samples for 72 h. After treatment, the cells were harvested and washed twice with phosphate-buffered saline (PBS), and the intercellular melanin was solubilized by 2 mL of 1 N NaOH and the collected samples were incubated at 60 °C for 1 h. The melanin content was determined by measuring the absorbance at 405 nm using a micro-plate reader (Biotek Instruments). The percentage of melanin content was calculated using the following formula: Inducer or Sample treatment group/control group × 100.

### 4.6. Protein Extraction and Western Blot Analysis

B16-F10 cells were seeded in to 10 cm cell culture dishes with a density of 1 × 10^6^ cell/dish. After an overnight incubation, cells were treated with melanogenesis inducer (a mixture of 20 μM FSK and 100 nM α-MSH) or various doses of test samples for 48 h. Cells were lysed by radio-immuno precipitation assay (RIPA) buffer (Pierce Biotechnology, Rockford, IL, USA). Protein concentration in the lysates were determined by Bio-Rad protein assay reagent (Bio-Rad Laboratories, Hercules, CA, USA). In total, 100 µg/lane of protein samples were separated by 10% SDS-PAGE. Then, the separated proteins were transferred onto polyvinylidene fluoride (PVDF) membrane for overnight. After the transfer, PVDF membranes were blocked with 5% skimmed milk for 90 min. After washed the membranes with TBST, the membranes were incubated with TYR, TRP-1, TRP-2, MITF, β-actin or GAPDH antibodies for overnight or 16 h, and then incubated with either HRP-conjugated anti-rabbit or anti-mouse antibodies for 2 h. Immunoblots were developed with the enhanced chemi-luminescence (ECL) reagents (Advansta Inc., San Jose, CA, USA), images were captured by ChemiDoc XRS+ docking system (Bio-Rad laboratories), and the protein bands were quantified by using Imagelab software (Bio-Rad laboratories).

### 4.7. GC–MS Analysis

To determine the chemical composition of CFEO, the GC-MS analyses using an ITQ 900 mass spectrometer coupled with a DB-5MS column as described previously [36] was conducted by us. The temperature program was as follows: 40 °C for 3 min, then increased to 3 °C/min to 180 °C, and then increased to 20 °C/min to 280 °C hold for 5 min. The other parameters were injection temperature, 240 °C; ion source temperature, 200 °C; EI, 70 eV; carrier gas, He 1 mL/min; and mass scan range, 40–600 *m*/*z*. The volatile compounds were identified by Wiley/NBS Registry of mass spectral databases (V. 8.0, Hoboken, NJ, USA), National Institute of Standards and Technology (NIST) Ver. 2.0 GC/MS libraries and the Kovats indices were calculated for all volatile constituents using a homologous series of *n*-alkanes C_9_-C_24_. The major components were identified by co-injection with standards (wherever possible).

### 4.8. Unknown Compounds Identification

The special compounds of CFEO, which could not be obtain by commercial supplier or previously studies, were purified and identified by chromatography and spectroscopy techniques in this study. CFEO was further separated by a HPLC by using the Agilent 1100 HPLC system equipped with a UV detector. A COSMOSIL C_18_-AR-II (10 mm I.D. × 250 mm, Nacalai Tesque Inc., San Diego, CA, USA) was employed with a MeOH/H_2_O solvent system. The gradient elution profile was as follows: 0 to 3 min, MeOH:H_2_O = 55: 45; 3 to 13 min, MeOH:H_2_O = 75:25; 13 to 18 min, MeOH:H_2_O = 75:25; 13 to 18 min, MeOH:H_2_O = 75:25; 18 to 25 min, MeOH:H_2_O = 85:15; 25 to 30 min, MeOH:H_2_O =85:15; 30 to 33 min, MeOH:H_2_O = 100:0; 5; 33 to 40 min, MeOH:H_2_O = 100:0 at a flowrate of 1.6 mL/min. The wavelength of the UV detector set at 254 nm. For structural identification, the compounds were dissolved in deuterated chloroform (d-chloroform; CDCl_3_) for detection, ^1^H, ^13^C, and 2D NMR spectra were recorded on a Bruker AVANCE III NMR spectrometer (Bruker, Billerica, MA, USA), acquiring ^1^H data at 400 MHz and ^13^C data at 100 MHz, using standard experiments from Bruker pulse programs library. High-resolution mass spectrometry (HR-MS) was determined using an LTQ Orbitrap XL (Thermo Fisher Scientific, Waltham, MA, USA). The spectral data of compounds **1** to **3** corresponded with a previous report and the obtained compounds were analyzed by MS and 400 MHz NMR. The spectral data of shonanic acid (**1**), thujic acid (**2**), and chaminic acid (**3**) [37,38] corresponded with a previous report.

Shonanic acid (**1**)

Colorless crystal; EIMS for C_10_H_14_O_2_ found 166; ^1^H-NMR (in CDCl_3_): δ(ppm) 1.13 (3H, s, H-10), 1.14 (3H, s, H-9), 2.48 (2H, t, *J* = 6.8 Hz, H-7), 3.43 (1H, m, H-1), 5.37 (1H, dq, *J* = 11.6, 1.4 Hz, H-5), 5.48 (1H, dq, *J* = 11.6, 4.4 Hz, H-3), 5.61 (1H, dq, *J* = 11.8, 6.0 Hz, H-6), 5.76 (1H, dq, *J* = 11.6, 4.4 Hz, H2).

Thujic acid (**2**)

Colorless crystal; EIMS for C_10_H_12_O_2_ found 164; ^1^H-NMR (in CDCl_3_): δ(ppm) 1.00 (3H, s, H-9), 1.00 (3H, s, H-10), 5.23 (1H, d, *J* = 10.0 Hz, H-3), 5.48 (1H, d, *J* = 9.6 Hz, H-5), 6.25 (1H, dd, *J* = 9.6, 6.8 Hz, H-6), 6.70 (1H, d, *J* = 10.4, 4.4 Hz, H-2), 7.64 (1H, d, *J* = 6.8 Hz, H-7).

Chaminic acid (**3**)

Colorless crystal; EIMS for C_10_H_14_O_2_ found 166; ^1^H-NMR (in CDCl_3_): δ(ppm) 0.68 (1H, m, H-1), 0.71 (3H, s, H-9), 0.83 (1H, m, H-6),1.04 (3H, s, H-8), 2.19 (2H, d, *J* = 17, H-5), 2.55 (2H, m, H-2), 7.01 (1H, s, H-3).

### 4.9. Statistical Analysis

Data are expressed as mean ± S.D of three independent experiments. Statistical analysis of the present study was performed using Graphpad Prism version 8.0 for Windows (GraphPad Software, La Jolla, CA, USA). Statistical significance was scored by using one-way ANOVA followed by Tukey’s test for multiple comparison. ^#^*p* < 0.01 was considered statistically significant for the α-MSH-FSK treatment vs. the control group. *p < 0.05, **p < 0.01, and ***p < 0.001 were considered statistically significant from sample treatment groups vs. α-MSH-FSK treatment group.

## 5. Conclusions

According to the results obtained in this study, we conclude that either CFEO or its active compound thymol could be a potential melanogenesis inhibitor. CFEO blocked melanin production in α-MSH-FSK-induced B16-F10 cells. The effects of CFEO on TRP-1 and TRP-2 might result from suppression of MITF transcriptional activity. The composition of CFEO was analyzed by GC-MS; the main components of CFEO were α-terpineol (23.47%), shonanic acid (10.45%), terpinen-4-ol (12.23%), thymol (5.3%), piperitone (3.44%), berbenone (2.81%), thujic acid (1.65%), and chaminic acid (0.13%). Moreover, shonanic acid (10.45%), thujic acid (1.65%), and chaminic acid (0.13%) were obtained through HPLC and identified by MS and NMR. These three compounds are uncommon ingredients in essential oils. Shonanic acid is a unique ingredient of CFEO, which can be used as an index component to identify CFEO. Among the composition of CFEO, thymol exhibited the strongest the inhibitory melanin production activity the anti-melanogenesis principal of CFEO might be thymol. Based on these results, we strongly suggest that CFEO could be a promising candidate for the development of skin whitening agents. However, further detailed investigation of this activity is necessary to elaborate the mechanisms and permit full exploitation of its skin whitening effects.

## Figures and Tables

**Table 1:** The major components and their relative contents (%) of wood essential oils of *Calocedrus formosana* (CFEO).

RT(min)	Compounds	Area (%)	KI	Identification
11.5	Camphene	1.11	951	MS KI ST
14.21	3-Carene	0.29	1009	MS KI ST
14.51	1,4-Cineol	0.82	1016	MS KI ST
14.94	*p*-*Cymene*	2.24	1026	MS KI ST
15.18	limonene	0.20	1031	MS KI ST
15.36	Eucalyptol (1,8-Cineol)	0.43	1035	MS KI ST
16.54	*trans*-Sabinenehydrate	0.17	1060	MS KI
17.61	1-Methylindan	0.54	1082	MS KI
17.83	α-Terpinolen	0.17	1086	MS KI ST
18.03	*p*-Cymenene	0.47	1090	MS KI ST
20.73	Camphor	1.89	1148	MS KI ST
22.34	Terpinen-4-ol	12.23	1181	MS KI ST
22.45	Myrtenol	1.18	1183	MS KI ST
23.02	α-Terpineol	23.47	1194	MS KI ST
23.55	Berbenone	2.81	1205	MS KI ST
25.71	Piperitone	3.44	1254	MS KI
26.79	*trans*-2-Caren-4-ol	1.36	1277	MS KI ST
27.75	Thymol	5.30	1296	MS KI ST
28.4	Carvacrol	0.47	1311	MS KI ST
29.29	Shonanic acid (**1**)	10.45	1332	MS ST
30.68	Perilla aldehyde	1.44	1364	MS KI ST
31.31	Thujic acid (**2**)	1.65	1378	MS ST
32.00	Chaminic acid (**3**)	0.13	1393	MS ST

KI: Kovats retention index on a DB-5MS column in reference to *n*-alkanes. ST: authentic standard compounds.MS, NIST and Wiley libraries and literature.

## Data Availability

All the raw data collected during this study are available upon request.
